# Experience a conflict—either consciously or not (commentary on Desender, Van Opstal, and Van den Bussche, 2014)

**DOI:** 10.3389/fpsyg.2015.00179

**Published:** 2015-02-19

**Authors:** Elger Abrahamse, Senne Braem

**Affiliations:** ^1^Department of Experimental Psychology, Ghent UniversityGhent, Belgium; ^2^Department of Experimental Clinical and Health Psychology, Ghent UniversityGhent, Belgium

**Keywords:** conflict adaptation, cognitive control, response conflict, consciousness, conflict monitoring

A general target of human cognition is to prevent or adapt to its own conflicts in information processing. Research on this issue employs conflict paradigms (e.g., Stroop task) in which salient task-irrelevant features either help (congruent trials) or hinder (incongruent trials) task-relevant processing. An important exercise within this domain is to obtain a clear understanding of the concept of conflict, and of the precise circumstances under which it arises and triggers behavioral adaptations. Throughout the last century various proposals have circulated (see Botvinick et al., 2001), but since Botvinick et al. (2001) proposed their seminal *conflict monitoring theory* the field has widely adopted “response conflict” as the main trigger for cognitive adaptations. Yet, in a recent paper, Desender et al. (2014) provided great fuel for discussion. Based on a simple but clever paradigm, they claim that not response conflict *per se* but rather *consciously experienced conflict* drives adaptation. Here we outline why Desender and colleagues overestimate the importance of their data in supporting a crucial role for conflict awareness, but underrate other aspects of their study.

Conflict monitoring theory was the first to offer a computational definition of cognitive conflict. Specifically, it proposes that anterior cingulate cortex (ACC; Carter and van Veen, 2007) tracks the total amount of energy at the response level: when multiple responses are simultaneously activated (response conflict), cognitive control needs to be enhanced. Thereby, the conflict monitoring theory formally disentangles “what happens on the screen” from “what happens in the brain.” Incongruent items generate substantial activation across different response nodes in the brain, and it is the subsequently generated conflict signal that triggers adapted information processing. Accordingly, congruency effects seem to be reduced following incongruent trials (i.e., *Gratton* effect; Gratton et al., 1992), a modulation also observed in ACC activity (Kerns et al., 2004).

By explicitly differentiating item (in)congruency and conflict, conflict monitoring theory opened up the possibility that—in principle—the two can be functionally dissociated, as sources other than item (in)congruency (see below) can contribute to conflict (and thus to adaptation) as well. Yet, although widely assumed across computational cognitive control models (Botvinick et al., 2001; Verguts and Notebaert, 2008; Jiang et al., 2014), Desender and colleagues are the first to provide an empirical foundation to this dissociation. In their study, participants performed a masked priming task in which barely visible primes could either be congruent or incongruent with the target. After each trial, participants reported whether or not they experienced conflict (irrespective of prime visibility). The crucial finding was that this subjective experience of conflict, but not item (in) congruency *per se*, determined conflict adaptation: the Gratton effect followed subjective report when it deviated from item (in) congruency. This indeed confirms that other sources than item (in) congruency drove conflict levels in the brain.

Interestingly, instead of fitting in with a stronghold of models on conflict adaptation, Desender et al. (2014) actually concluded against these models. Specifically, they claimed their data shows that “*the [conscious] experience of conflict, and not response conflict per se, is the crucial factor underlying cognitive adaptation effects*” (Desender et al., 2014, abstract). We feel that this conclusion—depicted in Figure 1A—is unnecessary because their data could equally well be captured by current conflict adaptation models. Specifically, models such as conflict monitoring theory allow quantifying the level of conflict, and larger conflict signals result in stronger conflict adaptation. At the same time, from the assumption that conscious experience is partially determined by signal strength (i.e., the stronger a signal, the higher the likelihood that it reaches consciousness; e.g., Cleeremans, 2008), larger conflict signals should also lead to a higher probability of subjective conflict experience. This indicates that larger conflict signals in the brain can drive both larger conflict adaptation *and* subjective experience *without* the latter two being causally related (Figure 1B). The study of Desender and colleagues does not imply a crucial role for conscious experience in conflict adaptation, as the link between them is purely correlational.

**Figure 1:**
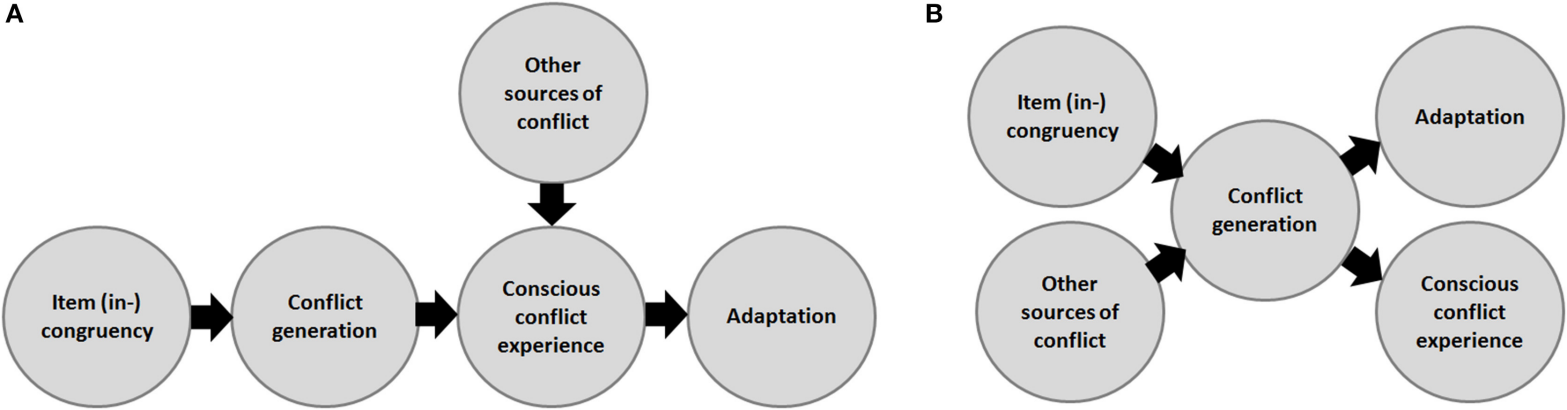
**Desender et al. (2014) are the first to show that sources other than item (in) congruency can contribute to conflict and subsequent adaptation**. Candidate sources may be responses biases (for example, through expectancies or response repetition effects), noise, or stimulus-response accidents such as when the letters “L” and “E” from a Stroop color-word like BLUE partly activate the response YELLOW due to letter sharing. Two scenarios may fit the data reported by Desender et al. (2014). **(A)** The scenario sketched by Desender and colleagues themselves, in which conscious conflict experience crucially modulates the link between conflict processing and adaptation. **(B)** An alternative scenario, consistent with current computational work on cognitive control, that construes conscious conflict experience and adaptation as two independent consequences of conflict processing in the brain. We believe that both scenarios are consistent with the data. Future work is required to empirically dissociate the two perspectives, and inspiration for this equally important as challenging research endeavor may be found, for example, in studies on the general role of expectancies in conflict adaptation (e.g., Duthoo and Notebaert, 2012; Jiménez and Méndez, 2013) and/or more direct measurements of conflict strength such as with electromyography recordings (e.g., Burle et al., 2005).

Why then did Desender and colleagues conclude otherwise? We believe a critical issue may be the authors' implicit assumption that additional sources contributing to conflict (like expectancies) can only work at the level of consious conflict experience. Instead, we would argue that expectancies (or any other type of response bias, such as residual activation as in response repetition effects, system noise, etc.), may equally well have a direct impact at the level of conflict generation (as modeled by Botvinick et al., 2001), before reaching conflict *awareness* (Figure 1B). Specifically, an overall bias toward a specific response will result in overall heightened activation at the response level. Therefore, conflict can occur even on congruent trials whenever these require a different response than the response that the system was biased toward. As such, the conclusion that “*subjective experience did [sometimes] not coincide with actual conflict*” (Desender et al., 2014; abstract) is not tenable, as due to additional sources at play, there is no way of measuring the *actual conflict* signal in the design of Desender and colleagues. Yet, this possibility—which would lead us to the perspective depicted in Figure 1B—was not considered by Desender and colleagues. An important future challenge will be to dissociate the two perspectives empirically.

Overall, Desender and colleagues demonstrate that multiple (simultaneous) sources can drive conflict generation in the brain. Interestingly, various computational models (Botvinick et al., 2001; Verguts and Notebaert, 2008) already tried to account for this by implementing noisy signals and residual activation from previous trials. The paradigm of Desender and colleagues probes future exploration of the precise sources of conflict besides item incongruency, and offers promising opportunities to cognitive neuroscientists interested in neural correlates of conflict processing and adaptation. However, in light of recent efforts to develop homunculus-free models of cognitive control (Verguts and Notebaert, 2009; Egner, 2014; Verbruggen et al., 2014), we want to remain extra cautious in assigning a crucial role to conscious conflict experience as long as the data do not directly necessitate this.

## Conflict of interest statement

The authors declare that the research was conducted in the absence of any commercial or financial relationships that could be construed as a potential conflict of interest.
